# Personality Traits and Postnatal Depression: The Mediated Role of Postnatal Anxiety and Moderated Role of Type of Birth

**DOI:** 10.3389/fpsyg.2019.01625

**Published:** 2019-07-12

**Authors:** Maria Roman, Cristina Maria Bostan, Loredana R. Diaconu-Gherasim, Ticu Constantin

**Affiliations:** Department of Psychology, Alexandru Ioan Cuza University, Iaşi, Romania

**Keywords:** personality traits, postnatal, depression, postnatal anxiety, type of birth, moderated mediation

## Abstract

This study investigated how personality traits are related to postnatal depression 2 weeks after giving birth and whether these relations are mediated by postnatal anxiety, measured after 3–4 days after giving birth and moderated by the type of birth. New mothers (*N* = 672, *M*age = 29.33) completed scales assessing their personality traits, postnatal anxiety, and postnatal depression 3 or 4 days after giving birth (T1). They also reported postnatal depression 2 weeks after giving birth (T2). Path analysis indicated that postnatal anxiety explained the link between personality traits (i.e., neuroticism) and postnatal depression 2 weeks after childbirth. The type of birth moderated the relation among, personality traits, postnatal anxiety and depression. Neuroticism and consciousness, in the natural birth’s group, and neuroticism and agreeableness, in the cesarean birth’s group, were associated with postnatal depression. Further, anxiety explained the relation between neuroticism and postnatal depression in both natural and cesarean birth groups. In addition, postnatal anxiety mediated the relation between extraversion and postnatal depression in the cesarean birth group. Our findings highlight that postnatal anxiety is a potential mechanism explaining how personality traits (i.e., neuroticism, extraversion) are related to postnatal depression, and that these relations may depend on the type of childbirth.

## Introduction

Several clinical phenomena for the postpartum period were described in the literature, including maternity blues, postnatal (or postpartum) depression and postnatal psychosis (Henshaw, 2003). The maternity blues (postnatal blues or third-day depression) period usually characterizes the first week after giving birth, and it is defined as a sad disposition that could be accompanied by affective dispositional lability, soft crying and confusion, fatigue, anxiety, insomnia, lost appetite and irritability (Clay and Seehusen, 2004; Robertson et al., 2004). The maternity blues symptoms had a maximum duration of 10 days and spontaneously and totally regressed, however, for approximately 20% of new mothers these symptoms can persist more and can evolve into postnatal depression (Teissedre and Chabrol, 2004; Reck et al., 2009). Thus, the clinical significance of baby blue symptoms could be seen in terms of the vulnerability associated with a higher probability of clinical depression in the postnatal period.

Postnatal depression is defined as a particular disorder of mood that may occur during the first year after giving birth, with a maximum incidence between 4 and 6 weeks after giving birth in any social, economic or cultural context (Stotland and Stotland, 1999; Munk-Olsen et al., 2006). Postnatal depression has harmful consequences on the well-being of mothers, and infants and relationships between mothers with the newborn and their partner (e.g., Grace et al., 2003; Dennis et al., 2004; Puckering, 2005). Considering the negative consequences with this disorder, more studies are needed to explore the role of psychological factors, which were less investigated in the previous literature (see Verkerk et al., 2005 for exceptions) on the etiology of postnatal depression. To advance the literature, our study explored how personality traits are linked to postnatal depression and whether postnatal anxiety and type of birth could be potential explaining mechanisms of these relations on a sample of new Romanian mothers.

### Personality Traits and Postnatal Depression

The term *personality traits* refers to a person’s psychological, behavioral or physical characteristics (Allport, 1961). Personality traits are relatively stable attributes of a person, a predisposition to respond in the same way to a variety of stimuli (Goldberg, 1990). According to the Big Five theory of personality (Costa and McCrae, 1992), there are five independent personality traits: neuroticism (tendency to suffer from psychological stress and inefficient coping strategies), extraversion (referring to the quantity and intensity of interpersonal relationships, activity, the need for stimulation and the ability to enjoy), opening up to experience (responsiveness to new situations), agreeableness (tendency to be empathetic, concerns about social harmony, optimistic opinions and the tendency to contradict negative emotional expressions), and conscientiousness (referring to the degree of organization, control and motivation). Previous literature suggests that one’s personality traits might reflect individual differences when it comes to reactivity to emotional and environmental cues (e.g., Corr, 2008, 2016; Hughes et al., 2012).

Among personality traits, neuroticism was consistently associated with emotional disturbances during the postnatal period. Specifically, cross-sectional studies have discovered that new mothers reporting high levels of neuroticism are more likely to experience higher levels of major postnatal depression or depressive symptomatology (e.g., Jones et al., 2010; Marín-Morales et al., 2014; Udovičić, 2014; Maliszewska et al., 2016). Fewer longitudinal studies explored the relation between neuroticism and postnatal depression, and contradictory findings were reported. One study found that high levels of neuroticism were associated with high levels of prenatal depression but were not related to postnatal depression (Kumar and Robson, 1984). Other studies showed that neuroticism was associated with high levels of postnatal depression over time (i.e., Boyce et al., 1991; Verkerk et al., 2005; Peñacoba-Puente et al., 2016).

Regarding extraversion, several cross-sectional and longitudinal studies found that women who reported high levels of extraversion also reported low levels of postnatal depression (e.g., Song et al., 2010; Maliszewska et al., 2016; Peñacoba-Puente et al., 2016), however, one study found that extraversion was not linked to postnatal depression (Martin-Santos et al., 2012).

A limited number of studies explored the relation between the other Big Five personality traits and postnatal depression and reported mixed findings. One study indicated that high levels of openness, agreeableness, or conscientiousness were associated with a lower risk of postnatal depression (Imširagić et al., 2014), whereas other studies found that only high levels of agreeableness (Song et al., 2010) or openness and conscientiousness (Udovičić, 2014) were linked with a lower risk of postnatal depression. Only two longitudinal studies explored how openness, agreeableness or conscientiousness are linked to subsequent postnatal depression, and the results indicated that these personality factors did not predict postnatal depression 1 year later (Verkerk et al., 2005; Peñacoba-Puente et al., 2016).

These inconsistent findings could be explained through the diversity of questionnaires used for assessing the personality traits (short form, Udovičić, 2014; long form, Peñacoba-Puente et al., 2016) or various ways of evaluating the participants’ depressive symptoms across studies (self-report scales, Imširagić et al., 2014; or clinical interview, Verkerk et al., 2005). These mixed findings point to the importance of additional studies to evaluate the relations between personality traits and postnatal depression, in order to better understand the strength and direction of the relations among these constructs.

### Personality Traits and Postnatal Anxiety

Despite the fact that previous literature reveals that postnatal anxiety disorders are more common than, or as frequent as, postnatal depression after delivery (e.g., Wenzel et al., 2005; Polachek et al., 2014), a limited number of studies investigated the link between personality traits and anxiety that occurs during the pregnancy period or in the first year after giving birth. In Peñacoba-Puente et al. (2016) study, women who reported high levels of neuroticism or low levels of extroversion and conscientiousness also reported high levels of prenatal anxiety, whereas agreeability and openness were not related with prenatal anxiety. Furthermore, previous studies consistently indicated that high levels of neuroticism (van Bussel et al., 2009; Conrad and Stricker, 2017) were linked to higher levels of anxiety after giving birth. The link between agreeableness and postnatal anxiety was less consistent; one study found that women who score higher on agreeableness also report more anxiety after giving birth (Conrad and Stricker, 2017), while another study found that agreeableness was not related with postnatal anxiety (van Bussel et al., 2009). Finally, extraversion, openness, and conscientiousness were not related to postnatal anxiety (van Bussel et al., 2009; Conrad and Stricker, 2017). These mixed results point to the importance of additional studies whose aim is to evaluate the relations between personality traits and postnatal anxiety.

### Postnatal Anxiety and Postnatal Depression

The tripartite theoretical model stipulates that anxiety symptoms are theoretically linked with depressive symptoms, suggesting overlapping in anxiety and depressive symptoms and comorbidity (Clark and Watson, 1991). This model states that anxiety and depression share a common component of negative affect (a significant non-specific component that encompasses general affective distress and other common symptoms), but these emotional disorders can also be distinguished by physiological hyper-arousal (specific to anxiety) versus the absence of positive affect (specific to depression). There is also empirical evidence supporting this relation on samples of pregnant woman (see Biaggi et al., 2016 for a review).

Further, an emerging body of research found that anxiety during pregnancy is an important risk factor for postnatal depression across various cultural contexts (see Norhayati et al., 2015 for a review; see also Alipoura et al., 2012; Peñacoba-Puente et al., 2016). These findings may be explained by the increased levels of anxiety sensitivity in depressed individuals, suggesting that anxiety could be associated with severity of depressive symptoms (Cox et al., 2001).

Previous literature emphasizes that a majority of pregnant women who experience clinical levels of anxiety, manifested postnatal anxiety later (Heron et al., 2004). However, there are differences between antenatal and postnatal anxiety from the point of view of characteristic fears; during pregnancy related anxiety fears refer to fear of labor and delivery, changes in one’s personal life and life style; changes in the relationship, while fears in about postnatal anxiety refer to separation fears from their child (van Bussel et al., 2009). In this context, more studies are needed to explore the relation between postnatal anxiety and postnatal depression over time.

### Postnatal Anxiety as a Mechanism Explaining the Relations of Personality Traits and Postnatal Depression

According to the hierarchical structure model (Mineka et al., 1998), personality traits are important vulnerability factors of various depressed manifestations. However, the empirical evidence for this idea is relatively limited (e.g., Murisa et al., 2005; Merino et al., 2016) and the mechanisms through which personality traits translate into the occurrence of the depressive symptoms are still relatively unclear (Barnhofer and Chittka, 2010).

A relatively small number of studies explored whether cognitive factors may explain these relations, and the results indicated that high levels of neuroticism were related with the high levels of worries, rumination or perceived stress, which in turn were linked to higher symptoms of depression (e.g., Murisa et al., 2005; Pereira-Morales et al., 2017). In addition, another study found that high levels of self-efficacy partially mediated the relations between personality traits (i.e., extraversion, agreeableness, conscientiousness, neuroticism) and depressive symptoms (Wang et al., 2014). A very limited number of studies investigated the mediating role of other explanatory mechanisms, as affective ones when it comes to these relations.

Previous literature shows that anxiety along with previous psychiatric illness, poor marital relationship and stressful life events are important predisposing factors of postpartum depression across various cultural contexts (see Norhayati et al., 2015 for review), thus suggesting that anxiety symptoms may potentially explain the associations between personality and postnatal depression. The studies investigating the role of anxiety for this relation are extremely limited. One such study investigated whether prenatal anxiety and depression may explain the relation between personality characteristics and postpartum syndromes, and the results showed that the links between dysfunctional perfectionism and avoidant personality styles and postnatal depression and anxiety were explained by prenatal depression and anxiety (Oddo-Sommerfeld et al., 2016). Second, the study explored the mediating role of prenatal anxiety on the relation between BigFive personality traits and postnatal depression, and the results indicated that high levels of neuroticism or low levels of extraversion were related to high levels of third-trimester symptoms of anxiety, which in turn were linked to high levels of postnatal depression (see Peñacoba-Puente et al., 2016 for an exception). It is important to mention that both of these studies explored the mediational role of antenatal anxiety in the relation between personality traits on postpartum syndromes.

The postnatal depression construct is conceptualized as a distinct construct from a major depressive disorder and includes unique symptoms, such as anxiety and loss of control (Norhayati et al., 2015). Previous studies found that the anxiety symptoms during pregnancy are a significant clinical risk factor for postnatal anxiety (Heron et al., 2004). Further, the highest prevalence rates of postnatal anxiety were reported in the first week after birth, while depression tends to occur later (e.g., Adewuya and Afolabi, 2005). Future studies are needed to better understand the role of postnatal anxiety in the relation between personality traits and postnatal depression.

### Birth Type as the Mechanism Explaining the Relation Between Personality Traits and Postnatal Anxiety and Depression

Previous literature reveals that women that give birth through a cesarean section procedure have a more negative perception over the experience of giving birth, a more negative self-image and manifest more anxiety in fulfilling their role of a mother compared to those giving birth through naturally (Lobel and DeLuca, 2007). However, the empirical studies provided inconsistent findings (see Xu et al., 2017 for meta-analysis). Some studies indicated that the type of birth did not impact the affective disorders during the postpartum period (e.g., Carter et al., 2006; Goker et al., 2012 for a review), whereas other studies indicated that the cesarean birth procedure could be considered as a more controlled and safe way of giving birth (e.g., Klint Carlander, 2014). On the other hand, other studies show that women who give birth through the cesarean section procedure have a higher risk of developing postpartum affective disposition compared to those that give birth through natural ways (see Borders, 2006; Olieman et al., 2017 for reviews). These findings were explained by the high levels of prenatal anxiety and depression that may increase the fear of childbirth and, consequently, from significant the cesarean predisposition (Storksen et al., 2012).

These contradictory findings could be explained by the individual differences in new mothers’ personality traits. Thus, some studies indicated, before birth, the women in the cesarean group were significantly higher in monotony avoidance and lower in socialization than those who did not request the procedure (Wiklund et al., 2006). Similarly, another study showed that extravagance and emotional stability were associated with the higher probability of natural birth (Johnston and Brown, 2013).

### The Present Study

To advance the literature, the first goal of the study was to explore the relation between personality traits and postnatal depression measured 2 weeks after giving birth. Based on previous literature (e.g., Song et al., 2010; Maliszewska et al., 2016), we expected that extraversion would be negatively related and neuroticism would be positively related with postnatal depression 2 weeks after giving birth. Further, we expected that agreeability, conscientiousness, and openness would be negatively related to postnatal depression.

The second goal of the study was to explore the relation between personality traits and postnatal anxiety. Based on previous findings (e.g., van Bussel et al., 2009; Conrad and Stricker, 2017), we expected that neuroticism and agreeableness would be positively related to postnatal anxiety. We did not formulate the hypothesis for the relation of openness, conscientiousness and extraversion with postnatal anxiety, because we did not have enough specific data on the meaning of these relations. Further, the third goal of this study was to explore the relation between new mothers’ postnatal anxiety assessed 3 to 4 days after giving birth and their levels of postnatal depression after 2 weeks of giving birth. Based on the previous literature showing that antenatal anxiety is a major risk factor for postnatal depression (e.g., Skouteris et al., 2009; Alipoura et al., 2012), we expected that postnatal anxiety would predict postnatal depression after 2 weeks.

Furthermore, we investigated whether the postnatal anxiety reported 3 to 4 days after giving birth, would explain the relation between personality factors and postnatal depression assessed 2 weeks after giving birth. Based on previous studies (Peñacoba-Puente et al., 2016) we expected that postnatal anxiety would be the explanatory mechanism for the relation between extraversion and neuroticism, rather than openness, conscientiousness and agreeableness, with postnatal depression. Finally, to our knowledge, no previous study has investigated whether the link between personality traits and postnatal affective disorders could be explained by the type of birth, as a moderator. To add to the literature on the subject, the fifth goal of this study we explored whether the type of birth would moderate the relation between personality traits as well as postnatal anxiety and depression.

It is important to mention that this work is a part of a longitudinal study investigating the role of personality traits on new mothers’ postpartum depression and how these characteristics interact with cognitive based interventions focusing on reducing the postpartum depression over time. We had two main goals: one about the relation between individual characteristics (i.e., personality traits) and development of symptoms of anxiety and depression, and second, about the efficiency of cognitive interventions on postpartum depression. We decided to measure the depressive symptoms after 12 days from birth, when the baby blue symptoms should have regressed (Reck et al., 2008) in order to capture the moment when these symptoms still persist and may evolve into postnatal depression. In other words, this moment had clinical significance in terms of the vulnerability associated with the higher probability of clinical depression occurring in the postnatal period.

Importantly, few previous studies evaluating the links between personality traits and emotions disturbance had been carried out on samples of new mother from Eastern European countries (see Imširagić et al., 2014 as an exception). Most studies were conducted in Western European societies (e.g., Wiklund et al., 2006; Marín-Morales et al., 2014). Further, the World Health Organization, United Nations Population Fund, and Key Centre for Women’s Health in Society (2009) recommends a cesarean frequency of 10%. However, the percentage of cesareans increases every year, especially in developing countries (Stanton and Holtz, 2006). These worrying statistics could be explained by the women’s phobia of birth pain and the fear of additional problems that may arise during birth delivery (Alipoura et al., 2012). In addition, an increasing number of physicians refuse to assist a natural birth, claiming the lack of time, the fear that something may go wrong, and everything will affect them (Khan, 2004). Thus, the women perceive natural birth as a forced situation rather than a natural phenomenon.

The percentage of cesarean surgery officially reached 47.2% in 2014 in Romania and around 60% unofficially reported from private clinics, coming in first place at the European level (Simionescu and Marin, 2017). Among all these cesarean surgeries, only 32.1% were justified as an emergency in labor. It is important to mention that, in our country the medical indications for cesarean section are not standardized at a national level, and there is no robust system to ensure the application of recommendations when it comes to using the cesarean section (Simionescu and Marin, 2017). In Romania, the health system is under-financed, and the National Health Insurance House allocates two to three times more money for cesarean section procedures compared to natural birth (depending on complications), and this motivates some hospitals to encourage cesarean sections as opposed to natural births. For these reasons we do not know the real percentage of planned versus emergency cesareans in our sample.

To enhance the literature, this short-term longitudinal study was conducted on a sample of Romanian new mothers. Thus, it is important to evaluate whether specific links among personality traits, postnatal emotional disturbance and the type of birth, found primarily in Western cultures, could be extended to an Eastern European cultural context.

Finally, previous literature showed that socio-demographic factors, such as age, education level and income, are risk factors for postnatal depression (see Norhayati et al., 2015 for a review). Specifically, low educational and low income levels were related with postnatal depression, being important for initial screening clinical evaluations (e.g., Mayberry et al., 2007; Buist et al., 2008). Based on these findings, we considered the role of area of residence, marital status and educational level on postnatal depression and to further control them when they appear as significant in the preliminary analysis.

## Materials and Methods

### Participants

A total of 719 new mothers (*M* = 28.26, *SD* = 4.6) from a medical facility in an urban area from the North-East of Romania after giving birth, were initially evaluated at 3–4 days after birth (Time 1) from 2016 to 2017. Adolescent mothers (<18 years) (*n* = 19), those with psychotic disorders (*n* = 2) and those with children born dead (*n* = 3) were not included in the study. Among all new mothers, only 672 new mothers mean age = 29.33 (*SD* = 5.44, range = 18–43 years completed the depression scale after 2 weeks following birth (Time 2). Most new mothers (63.8%) reported living in an urban area and 36.2% living in a rural area. Of all the participants, 15.5% of the women had elementary education, 43.6% had completed secondary education, and 40.9% stated that they had a college degree or higher. In addition, 83% of the women reported being married and 17% were single mothers (divorced or separated). Out of the total number of the participants, 46.1% gave birth through natural ways, and 53.9% gave birth through the cesarean section procedure.

### Procedure

The IRB was obtained before beginning the study, and we followed the ethical principles for research on human participants described in the Declaration of Helsinki. We also obtained an institutional approval from the ethic committee of the maternity hospital and written informed consent from the women for voluntary participation. Initially, the women completed questionnaires regarding personality traits, postnatal anxiety, and depression 3 or 4 days after giving birth (T1). They also filled in a demographic questionnaire (e.g., age, educational level, area of residence, marital status, type of birth). All the participants completed these scales using the data pencil-paper method during T1. Two weeks after giving birth (T2), when baby blue symptoms should spontaneously regress, the new mothers filled in a questionnaire assessing postnatal depression, via e-mail.

### Measures

#### Personality Traits

We used the Big Five^©^plus personality inventory (Constantin et al., 2010), built upon the BigFive model (Goldberg et al., 2005) to assess the personality traits. This instrument consists of 240 items evaluating five traits of personality: extraversion [48 items, α = 0.89; e.g., When I am at a party: (a) I am *at* the center of attention or (b) I *would* rather stay in the background], agreeableness [48 items, α = 0.76; e.g., My professional life is dominated by decisions that: (a) bring me personal benefits or promotion opportunities and (b) contribute to maintaining good relationships with others], neuroticism [48 items, α = 0.87; e.g., When I think about my past, I see: (a) pleasant and happy events or (b) unpleasant and sad events], conscientiousness [48 items, α = 0.83; e.g., You can say about me that: (a) I usually delay my financial or moral payments or (b) I strictly respect my commitments or the payments I have to do] and openness [48 items, α = 0.76; e.g., My personal preferences usually are: (a) practical and useful or (b) artistic and esthetic]. We computed the sum across the items for each personality trait. Previous studies indicated good psychometric properties, and validity showed good internal consistency and test-retest fidelity (Constantin et al., 2010).

#### Postnatal Depression

Edinburgh Postnatal Depression Scale (EPDS; Cox et al., 1987) was used to assess postnatal depression. The scale consists of 10 questions measuring postnatal depression symptoms (α = 0.85 and 0.79 at T1 and T2, respectively, e.g., I have been so unhappy that I have been crying). Items were rated from 0 (not at all or never) to 3 (yes, all the time or very often). A total sum score was computed.

#### Postnatal Anxiety

The Hamilton Anxiety Rating Scale (HARS; Hamilton, 1959) was used to evaluate the severity of the anxiety symptoms. The 14-item scale identifies the psychological and somatic symptoms of anxiety and how severe they are after birth (α = 0.83, e.g., Fear of the dark, of strangers, of being left alone, of animals, of traffic, of crowds). The participants responded to each item using a 4-point Likert scale varying from 0 (absent) to 4 (very severe). A total sum score was computed.

### Data Analytic Plan

We initially provided descriptive data regarding women’s levels of postnatal depression, postnatal anxiety and type of delivery, and then we explored the relations between demographic variables and postnatal depression (T2). Zero-order correlations among the main study variables were computed on the entire sample and also separately for each type of birth, The differences regarding the associations between constructs for each type of birth were also explored using a Fisher’s *r* to *Z* transformation. We used IBM SPSS Statistics 21. Further, we tested the role of postnatal anxiety as a mediator for the relation between personality traits and postnatal depression for the entire sample using the structural equation modeling (SEM) and multiple group SEM in order to test the mediation of postnatal anxiety for each type of birth group (Hair et al., 2010). We used AMOS IBM 21.0 version for SEM analysis. The model fit for each model was assessed based on a Chi-square, the (GFI > 0.90), Tucker Lewis Index (TLI > 0.95), comparative fit index (CFI > 0.95) and root mean square error of approximation (RMSEA < 0.06 to 0.08) (Schreiber et al., 2006). We included covariances among the variables (e.g., covariances between personality traits, between personality traits, initial postnatal depression). The participants’ educational level and initial postnatal depression was assessed 3–4 days after giving birth (T1) were entered in the final model as control variables for postnatal depression assessed after 2 weeks. We used bootstrapping techniques to test the significance of direct effects of personality traits, indirect effects through postnatal anxiety and total effects of personality traits and postnatal anxiety over postnatal depression for each subgroup (Byrne, 2016). We used a sample of 5,000 for bootstrapping and a 95% Confidence Interval, where the absence of zero indicates a significant effect (Preacher and Hayes, 2008). In addition, we used a critical ratio to assess the significance of direct effects moderation by type of birth (Lowry and Gaskin, 2014). Finally, we used the heterogeneity test, as recommended by Altman and Bland (2003), in order to assess if there are significant differences between the unstandardized estimates of indirect effects of each group to test for the moderated effects.

## Results

### Preliminary Analysis

The descriptive analysis showed that in our study, 24.3% of women had a score above 14 at postnatal anxiety, indicating relevant and clinical levels of postnatal anxiety (Hamilton, 1959). Further, 28% of the women obtained a score at postnatal depression above the cutoff point of 12 points (Cox et al., 1987) after 3 or 4 days after giving birth and 20% of the women after 2 weeks, which indicates relevant and clinical levels of postnatal depression. In addition, 46.1% of the new mothers gave birth through natural ways and 53.9% gave birth through the cesarean section procedure.

The *t*-test indicated that there were no residence area or marital status differences on postnatal depression at T1 and T2, *ts*(670) < 1,79, all *ps* > 0.13. There was no main effect of the women’s educational levels on their postnatal depression at T1, *F*(2,669) = 3.01, *p* > 0.05. We found a main effect of educational level on postnatal depression at T2, *F*(2,669) = 7.38, *p* = 0.001*;* women with elementary education reported higher levels of postnatal depression (*M* = 8.17, *SD* = 5.78) than women with high school or college degree (*M* = 5.69, *SD* = 2.00 and *M* = 4.66, *SD* = 3.6, respectively). Further, zero-order correlations indicated that the participants’ age was significantly negatively associated with postnatal depression, measured at T1 and T2 (*r*s = -0.14 and -0.15, respectively, all *ps* < 0.001).

The *t*-test indicated that there were no birth type differences on postnatal depression (T1) measured 3 or 4 days after giving birth, *t*(670) = 0.58, *p* = 0.55. Further, there were birth type differences for postnatal depression T2, *t*(670) = 1.99, *p* = 0.04; women who gave birth naturally reported higher levels of postnatal depression (*M* = 6.15, *SD* = 0.34) compared to those who underwent the cesarean procedure (*M* = 5.23, *SD* = 0.31).

### Correlation Analysis Between Personality Traits, Postnatal Anxiety, and Postnatal Depression

Zero-order correlations among personality traits, postnatal anxiety and depression for the entire sample are shown in Table 1. Extraversion and conscientiousness were significantly negatively related to postnatal depression, whereas neuroticism was positively associated with postnatal depression for the entire sample at both T1 and T2 (see Table 1). Agreeableness and openness were not significantly related to postnatal depression at T1 and T2 for the entire sample. Extraversion and conscientiousness significantly negatively correlated with postnatal anxiety at T1 and T2. Further, postnatal anxiety was significantly positively associated with postnatal depression for both T1 and T2 for the entire sample.

**Table 1:** Zero-order correlations between personality traits, postnatal anxiety and postnatal depression for the entire sample.

	*M*	*SD*	Minimum	Maximum	1	2	3	4	5	6	7
(1) Extraversion T1	25.15	9.45	1	47							
(2) Agreeableness T1	29.32	6.46	4	46	0.20**						
(3) Neuroticism T1	20.72	8.69	1	44	-0.34**	-0.15**					
(4) Conscientiousness T1	28.23	7.72	3	47	0.15**	0.10**	-0.43**				
(5) Openness T1	19.96	6.47	2	41	0.33**	0.01	-0.18**	-0.18**			
(6) Postnatal anxiety T1	8.10	8.93	0	45	-0.14**	-0.05	0.33**	-0.14**	-0.02		
(7) Postnatal depression T1	8.06	5.75	0	29	-0.20**	-0.07	0.36**	-0.20**	-0.01	0.71**	
(8) Postnatal depression T2	5.65	5.98	0	29	-0.18**	-0.04	0.36**	-0.22**	-0.01	0.74**	0.84**

*N = 672. ^∗^*p* < 0.05; ^∗∗^*p* < 0.001*.

Similar associations were also found between personality traits, postnatal anxiety, and depression for both T1 and T2 for each type of birth group (see Table 2).

**Table 2:** Zero-order correlations between personality traits, postnatal anxiety and postnatal depression for each type of birth.

Variables	*M*	*SD*	Minimum	Maximum	1	2	3	4	5	6	7	8	*M*	*SD*	Minimum	Maximum
(1) Extraversion T1	23.52	9.08	1	46		0.22**	-0.32**	0.09	0.36**	-0.13*	-0.24**	-0.16**	26.54	9.54	4	47
(2) Agreeableness T1	28.92	6.50	10	46	0.16**		-.14**	0.06	0.05	-0.05	-0.09	-0.01	29.67	6.42	4	46
(3) Neuroticism T1	20.66	8.25	1	44	-0.36**	-0.17**		-0.38**	-0.19**	0.35**	0.41**	0.40**	19.93	9.06	1	41
(4) Conscientiousness T1	27.41	7.62	3	45	0.18**	0.13*	-0.48**		-0.20**	-0.13**	-0.21**	-0.19**	28.93	7.74	7	47
(5) Openness T1	19.82	6.36	2	41	0.29**	-0.05	-0.17**	-0.16**		-0.003	0.03	0.03	20.07	6.57	3	41
(6) Postnatal Anxiety T1	8.61	0.51	0	45	-0.14*	-0.05	0.30**	-0.14*	-0.05		0.71**	0.76**	7.66	9.05	0	40
(7) Postnatal depression T1	8.20	0.33	0	29	-0.15**	-0.04	0.30**	-0.19**	-0.07	0.70**		0.82**	7.94	6.04	0	28
(8) Postnatal depression T2	6.15	0.34	0	29	-0.19**	-0.07	0.30**	-0.23**	-0.05	0.72**	0.86**		5.23	5.89	0	23

*Zero-order correlations for the cesarean birth group (*n* = 362) are presented above the diagonal blank line, and those for the natural birth group (*n* = 310) are presented below the diagonal blank line. *^∗^p* < 0.05; *^∗∗^p* < 0.001*.

We also tested whether there is a significant type of birth (natural vs. cesarean section) difference between natural regarding the straights of correlations between constructs, using the Eid et al. (2017) procedure. The results indicated no significant birth type difference (*Zs* < 1.39, all *ps* > 0.05).

### Path Analysis Testing the Study Hypotheses

**Figure 1:**
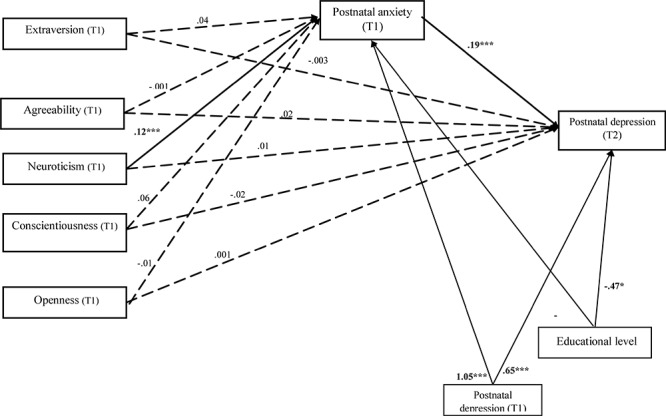
Unstandardized path estimates of the mediation model. The continuous lines indicate statistically significant relations and the dotted lines indicate non-significant relations between constructs. ^∗^*p* < 0.05; ^∗∗^*p* < 0.01; ^∗∗∗^*p* < 0.001.

Further, we tested whether postnatal anxiety may explain the relation between personality traits and postnatal depression. The results showed that the model has a good fit with the data for the entire sample: χ^2^ = 62, df = 9, *p* < 0.001, GFI = 0.98, CFI = 0.97, TLI = 0.89 and RMSEA = 0.08, the model explaining 75.9% of postnatal depression. Unstandardized path estimates are shown in Figure 1, and detailed results of direct, indirect and total effects are presented in Table 3. Personality traits did not predict postnatal depression directly. Further, the results showed that only neuroticism directly predicted postnatal anxiety (*B* = 0.12, *p* < 0.001) for the entire sample. Postnatal anxiety was positively significantly related to postnatal depression (β = 0.28, *p* < 0.001). Regarding the control variable, educational level negatively and significantly predicted postnatal anxiety at T1 (*B* = -1.18, *p* < 0.001) and postnatal depression at T2 (*B* = -0.47, *p* = 0.03). Postnatal depression at T1 significantly positively predicted postnatal depression after 2 weeks (*B* = 0.65, *p* < 0.001).

**Table 3:** Mediation effects of postnatal anxiety in the association between personality traits and postnatal depression at T2 for the entire sample.

Parameter	Estimate (S.E.)	Lower	Upper
**Standardized direct effects**			
PD (T2) < --- Extraversion	-0.005 (0.02)	-0.049	0.036
PD (T2) < --- Agreeableness	0.02 (0.02)	-0.014	0.066
PD (T2) < --- Neuroticism	0.02 (0.02)	-0.023	0.071
PD (T2) < --- Conscientiousness	-0.03 (0.02)	-0.082	0.011
PD (T2) < --- Openness	0.001 (0.02)	-0.041	0.040
PD (T2) < --- Postnatal anxiety	0.28^∗∗∗^ (0.03)	0.223	0.348
**Standardized indirect effects**			
PD (T2) < --- postnatal anxiety < --- Extraversion	0.01 (0.008)	-0.003	0.030
PD (T2) < --- postnatal anxiety < --- Agreeableness	-0.001 (0.008)	-0.016	0.014
PD (T2) < --- postnatal anxiety < --- Neuroticism	0.03^∗∗^ (0.01)	0.013	0.055
PD (T2) < --- postnatal anxiety < --- Conscientiousness	0.01 (0.01)	-0.002	0.036
PD (T2) < --- postnatal anxiety < --- Openness	-0.002 (0.009)	-0.020	0.015
**Standardized total effects**			
PD (T2) < --- Extraversion	0.008 (0.02)	-0.039	0.053
PD (T2) < --- Agreeableness	0.02 (0.02)	-0.017	0.067
PD (T2) < --- Neuroticism	0.05^∗∗^ (0.02)	0.006	0.107
PD (T2) < --- Conscientiousness	-0.02 (0.02)	-0.069	0.032
PD (T2) < --- Openness	0.002 (0.02)	-0.049	0.042
PD (T2) < --- postnatal anxiety	0.28^∗∗∗^ (0.03)	0.223.	0.348

*PD (T2) = postnatal depression after 2 weeks, S.E. = standard error. Lower and upper limits of the confidence intervals used to assess the significance of direct, indirect and total effects are presented in vertical columns in the right part of the table. ^∗∗^*p* < 0.05; ^∗∗∗^*p* < 0.001*.

The bootstrapping technique indicated whether personality traits were related to postnatal depressive symptoms through postnatal anxiety (see Table 3). There was a significant indirect effect of neuroticism over postnatal depression (β = 0.03, *p* = 0.002, CI 95% [0.013, 0.055], indicating an increase of postnatal depression after 2 weeks, through postnatal anxiety. The total effects of neuroticism with postnatal anxiety, over postnatal depression was significant (β = 0.05, *p* = 0.002, CI 95% [0.006, 0.107]). The results indicated a full mediation role of postnatal anxiety for the relation between neuroticism and postnatal depression. No other indirect effects were significant between personality traits and postnatal depression after 2 weeks, when controlling for postnatal anxiety.

### The Multi-Group Structural Equation Modeling Analysis for Testing the Moderated Mediation Relation Between Personality Traits and Postnatal Depression Through Postnatal Anxiety for Each Type of Birth Group

**Figure 2:**
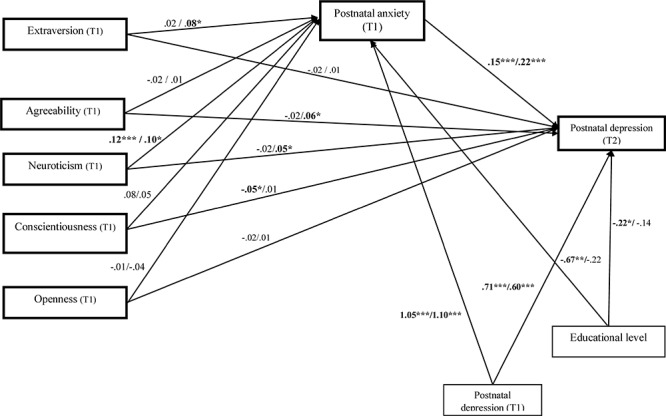
Unstandardized path estimates of the multi-group mediation model. From left to right, the unstandirdized coefficients for the natural birth group were presented firth and then for the cesarean procedure type of birth group. Postnatal depression (T1) = postnatal depression after 3 or 4 days, Postnatal depression (T2) = postnatal depression after 2 weeks. Postnatal anxiety (T1) = postnatal anxiety after 3 or 4 days. ^∗^*p* < 0.05; ^∗∗^*p* < 0.01; ^∗∗∗^*p* < 0.001.

The multi-group structural equation modeling analysis simultaneously tested the relations between personality traits, postnatal anxiety and postnatal depression, as well as the mediated role of postnatal anxiety for each subgroup of birth type. In the natural type of birth group, the fit indices showed that the model has a good fit with the data, χ^2^ = 19.52, *df* = 18, *p* = 0.06, GFI = 0.95, CFI = 0.99, TLI = 0.96, and RMSEA = 0.03. The model explained 75.5% of variance of postnatal depression 2 weeks after giving birth. Unstandardized estimates are presented in Figure 2.

The detailed results of direct, indirect and total effects are presented in Table 4. The bootstrapping method indicated that in the natural type of birth group, only conscientiousness significantly predicted postnatal depression at T2 (β = -0.06, *SE* = 0.02, *p* = 0.03). Further, neuroticism significantly predicted postnatal anxiety (β = 0.12, *SE* = 0.05, *p* = 0.02). Postnatal anxiety at T1 positively predicted postnatal depression after 2 weeks at T2 (β = 0.21, *SE* = 0.26, *p* < 0.001). Finally, the educational level predicted postnatal anxiety (*B* = -0.67, *p* = 0.02). The educational level (*B* = -0.22, *p* = 0.02) and initial postnatal depression (T1) predicted new mothers’ postnatal depression after 2 weeks (T2) (*B* = -0.71, *p* < 0.001).

**Table 4:** Direct and indirect effects and 90% confidence intervals.

	Model (A): natural			Model (B): cesarean		
Parameter	Estimate	Lower	Upper	Estimate	Lower	Upper
**Standardized direct effects**						
PD (T2) < --- Extraversion	-0.03 (0.02)	-0.082	0.012	0.02 (0.03)	-0.032	0.074
PD (T2) < --- Agreeableness	-0.02 (0.02)	-0.072	0.022	0.06 (0.02)**	0.021	0.113
PD (T2) < --- Neuroticism	0.03 (0.03)	-0.093	0.023	0.06 (0.03)**	0.015	0.118
PD (T2) < --- Conscientiousness	-0.06 (0.03)**	-0.120	-0.009	-0.006 (0.03)	-0.058	0.044
PD (T2) < --- Openness	0.02 (0.04)	-0.068	0.033	0.01 (0.03)	-0.038	0.062
PD (T2) < --- postnatal anxiety	0.21 (0.04)***	0.143	0.290	0.33 (0.04)***	0.253	0.407
**Standardized indirect effects**						
PD (T2) < --- postnatal anxiety < --- Extraversion	0.004 (0.01)	-0.012	0.021	0.02 (0.01)**	0.007	0.051
PD (T2) < --- postnatal anxiety < --- Agreeableness	-0.004 (0.008)	-0.019	0.009	0.001 (0.01)	-0.019	0.023
PD (T2) < --- postnatal anxiety < --- Neuroticism	0.02 (0.01)**	0.006	0.050	0.03 (0.01)**	0.012	0.063
PD (T2) < --- postnatal anxiety < --- Conscientiousness	0.01 (0.01)	-0.001	0.035	0.01 (0.01)	-0.011	0.040
PD (T2) < --- postnatal anxiety < --- Openness	-0.001 (.01)	-0.020	0.016	-0.009 (0.01)	-0.033	0.013
**Standardized total effects**						
PD (T2) < --- Extraversion	-0.03 (0.02)	-0.079	0.018	0.04 (0.03)	-0.010	0.104
PD (T2) < --- Agreeableness	-0.02 (0.03)	-0.079	0.020	0.06 (0.03)**	0.019	0.117
PD (T2) < --- Neuroticism	0.01 (0.03)	-0.071	0.053	0.10 (0.03)**	0.047	0.159
PD (T2) < --- Conscientiousness	-0.05 (0.03)	-0.107	0.008	0.007 (0.03)	-0.051	0.064
PD (T2) < --- Openness	-0.01 (0.03)	-0.075	0.033	0.005 (0.03)	-0.049	0.059
PD (T2) < --- Postnatal anxiety	0.21 (0.04)	0.143	0.290	0.33 (0.04)	0.253	0.407

*PD (T2) = postnatal depression after 2 weeks. Model (A): natural = the natural birth group; Model (B): cesarean = the cesarean birth group. *^∗∗^p* < 0.05; *^∗∗∗^p* < 0.001*.

We also identified a significant positive indirect effect (β = 0.02, *SE* = 0.01, *p* = 0.02, CI 95% [0.006, 0.050] between neuroticism and postnatal depression at T2 through postnatal anxiety. The results support full mediation regarding the role of anxiety as a mediator for the relation between neuroticism trait and postnatal depression. For the cesarean birth group, neuroticism significantly predicted postnatal anxiety (β = 0.10, *SE* = 0.03, *p* = 0.03). Further, neuroticism significantly predicted postnatal depression at T2 (β = 0.06, *SE* = 0.03, *p* = 0.04, CI 95%, [0.015, 0.118]) (see Figure 2). Higher agreeableness positively directly predicted postnatal depression (β = 0.06, *SE* = 0.02, *p* = 0.01, CI 95%, [0.021, 0.011]). No direct and significant effects were observed from extraversion, conscientiousness and openness over postnatal depression after 2 weeks. Mediation analysis (see Table 4) indicated a significant indirect effect of extraversion on postnatal depression (β = 0.02, *SE* = 0.01, *p* = 0.03, CI 95%, [0.007, 0.051]) through postnatal anxiety, supporting a full mediation effect. Regarding neuroticism, the results showed an indirect effect, which was significant after controlling for postnatal anxiety (β = 0.03, *SE* = 0.01, *p* = 0.01, CI 95%, [0.012, 0.063]), indicating partial mediation. The total effect of neuroticism and postnatal anxiety is higher and more significant than the direct effect (β = 0.10, *SE* = 0.03, *p* = 0.005, CI 95%, [0.046, 0.161]). Agreeableness, conscientiousness and openness had no significant effect after controlling for postnatal anxiety, indicating no mediation.

We also tested whether the type of birth moderates the associations between personality traits and postnatal depression. The results of critical ratios pairwise parameter comparisons support a moderating role of birth type for the direct effect of agreeableness over postnatal depression after 2 weeks (*Z* = 2.47, *p* < 0.001). There was a significant direct effect for the women who gave birth through the cesarean section (*b* = 0.06, *p* = 0.009) but not for women from the natural birth group (*b* = -0.02, *p* = 0.36). The results also supported the type of birth as a moderator for the direct effect of neuroticism on postnatal depression after 2 weeks (*Z* = 1.97, *p* < 0.001); there was a significant direct effect for women from the cesarean group (*b* = 0.04, *p* = 0.04) but not for women from the natural birth group (*b* = 0.02, *p* = 0.38). The type of birth did not moderate the direct relation of extraversion, conscientiousness and openness with postnatal depression after 2 weeks (*Zs <* 0.55, *ps* > 0.05).

Finally, we tested the moderated indirect effects for each birth type group using the unstandardized estimates. The results of the heterogeneity test support a moderating role of birth type for the impact of extraversion over postnatal depression (Z = 2.01, *p* = 0.04) and for the indirect effect of postnatal anxiety; the indirect effect was significant for the cesarean birth group (unstandardized *B* = 0.02, *SE* = 0.008, *p* = 0.03) but not for the natural birth group (unstandardized *B* = 0.001, *SE* = 0.005, *p* = 0.67). The type of birth did not moderate the indirect effects of agreeableness, neuroticism, conscientiousness and openness on postnatal depression after 2 weeks through postnatal anxiety (*Zs* < 0.54, *ps* > 0.05).

## Discussion

This study explored how personality traits are related to postnatal depression, and whether postnatal anxiety, measured immediately after birth, might explain the relation between personality traits and postnatal depression. In addition, we tested whether the relations between personality traits, postnatal anxiety, and depression are moderated by type of birth.

In line with previous literature (e.g., Jones et al., 2010; Marín-Morales et al., 2014; Udovičić, 2014; Maliszewska et al., 2016), high levels of neuroticism and low levels of extraversion and consciousness were associated with high levels of postnatal depression. Agreeableness and openness were not significantly associated with postnatal depression 2 weeks after giving birth, and these findings are concordant with previous studies (e.g., Verkerk et al., 2005). However, the results of a path analysis of the direct effect model, including personality traits simultaneously and taking into account the effects of the demographic variables, indicated that personality traits do not have a unique and independent contribution to postnatal depression after 2 weeks. Similar results were found in another previous study (Peñacoba-Puente et al., 2016).

Despite empirical links between personality traits and postnatal depression, the question of how to explain these associations received little attention in the literature. In this study, we evaluated whether postnatal anxiety may explain these relations. At the first step, we examined how personality traits are related to new mothers’ postnatal anxiety. In line with previous studies (e.g., van Bussel et al., 2009; Peñacoba-Puente et al., 2016; Conrad and Stricker, 2017), our findings showed that new mothers with high levels of neuroticism and low levels of extraversion or consciousness also reported higher postnatal anxiety, whereas agreeableness and openness were not related with postnatal anxiety. The results of the path analysis, simultaneously including personality traits, indicated that only neuroticism has a unique and independent contribution to postnatal anxiety when considering the entire sample. These findings are in line with previous literature (van Bussel et al., 2009; Conrad and Stricker, 2017).

Further, correlational and path analysis indicated that the new mothers with high levels of postnatal anxiety reported 3 or 4 days after birth also reported higher levels of postnatal depression after 2 weeks. These findings are consistent with the hierarchical structure model of depression (Mineka et al., 1998) and similar to the findings of previous studies also indicating a relation between these constructs during pregnancy and postnatal periods (Alipoura et al., 2012; Peñacoba-Puente et al., 2016).

Our study advances the literature investigating whether postnatal anxiety might explain the relations between personality traits and postnatal depression. Our results indicate that postnatal anxiety fully mediates the relation between neuroticism (rather than extraversion, agreeableness, openness or consciousness) and postnatal depression assessed 2 weeks after giving birth. Specifically, new mothers reporting high levels of neuroticism also reported higher levels of postnatal anxiety 3–4 days after giving birth, which in turn was related to postnatal depression 2 weeks from giving birth. These findings are consistent with Peñacoba-Puente et al. (2016) study, which also shows that the relation between neuroticism and postnatal depression was explained by third-trimester anxiety symptoms (Peñacoba-Puente et al., 2016). These findings could be further explored in future studies by empirically testing models including other personality characteristics, such as locus of control (Moshki et al., 2014), relevant to postnatal depression. Further, our work could also be extended by evaluating the other potential mediating roles of other (affective or cognitive) factors (e.g., parenting stress, antenatal anxiety, depression) in order to further understand the complex relations among personality traits and postnatal anxiety and depression.

Previous literature reveals links between personality traits and the type of birth, as well as between the type of birth and postnatal anxiety and depression (e.g., Goker et al., 2012).On the other hand, it was not previously explored whether the type of birth could explain the link between personality traits and postnatal anxiety and depression. In this study, we evaluated whether the type of birth (natural birth vs. cesarean section) may explain the relations among these constructs. Initially, we examined whether the relation between personality traits and whether postnatal anxiety is moderated by the type of birth. The path analysis indicated that, in the natural births’ group, neuroticism was significantly positively associated with postnatal depression, while consciousness was negatively associated. In the cesarean birth group, neuroticism and agreeableness were directly and positively related to postnatal depression. Critical ratio pairwise comparisons indicated significant differences between groups only for the relations of agreeableness and neuroticism with postnatal depression: these relations were significant only for the women from the cesarean section group, compared to those from the natural birth group.

These findings could be explained by the fact that women requesting a cesarean section, without a specific medical indication, may display anxious feelings, a lack of confidence and fear of giving birth (Zhao and Chen, 2013). Further, they also may experience loss of self-esteem, more feelings of failure and higher self-blame and, consequently, they have a higher risk for developing postpartum affective disposition compared to those that give birth through natural ways (see Borders, 2006 for review). It is important to mention that in our country there are no standardized protocols containing the medical indications for cesarean section and cesarean sections as opposed to natural births, which are encouraged because they are better financed by the National Health Insurance House (Simionescu and Marin, 2017). Thus, we could not make a real difference between emergency and planned cesareans, so we cannot compare the relations among our study’s constructs based on type of cesarean section. In addition, these results could be explained through differences in personality characteristics between women giving birth naturally compared to those who underwent the cesarean section (Wiklund et al., 2006); women from the cesarean group reported higher neuroticism (i.e., lower emotional stability) and than those who did not request the procedure (Johnston and Brown, 2013).

Furthermore, the types of birth groups did not moderate the exampling role of anxiety symptoms in the relation between neuroticism and postnatal depression. In both groups, high levels of neuroticism were linked to higher postnatal anxiety which in turn is related to higher depressive symptoms. These results add to the previous literature showing the indirect effect of neuroticism on postnatal depression through anxiety, regardless of the type of birth.

Further, we found a moderating role of the type of birth for the mediating role of anxiety for the link between extraversion and postnatal depression. This relation was relevant for the cesarean birth group but not for the natural birth group. Thus, high levels of extraversion were related to high levels of postnatal depression, when women from the cesarean group experienced high levels of postnatal anxiety. These findings indicate that extraversion could be a risk factor for postnatal depression because of the positive correlation with anxiety symptoms in cesarean conditions. These results contradict previous literature suggesting that low extraversion is linked with anxiety disorders on samples of adults (e.g., Bienvenu et al., 2001; Gomez and Francis, 2003). Further, these findings are in line with the Peñacoba-Puente et al. (2016) study, which also reports that the link between extraversion and postnatal depression was explained by prenatal anxiety symptoms, but in the opposite direction. In their study, low levels of extraversion were related to high levels of prenatal anxiety, which in turn was linked to high levels of postnatal depression. The differences between our study and Peñacoba-Puente et al. (2016) study could be explained through the time interval between the measurements: in our study, the personality traits and anxiety were assessed 3–4 days after childbirth, while in Peñacoba-Puente et al. (2016) study the personality traits and anxiety were evaluated during the prenatal period (first and third trimester).

A possible explanation would be the stronger relation between extraversion and postnatal depression in a sample of women from the cesarean birth group who experienced a higher risk for developing postpartum affective disposition, compared to those from the natural birth group, due to their experience of increasing loss of self-esteem and self-blame as well as more feelings of failure (see Borders, 2006 for review). However, these findings are in line with trait congruence theory (Rusting, 1999), suggesting that people with high scores in extraversion tend to use automatic emotional regulation strategies based on their need to validate their personal self. Previous studies showed that people with high scores on extraversion prefer to display positive emotions and to make positive judgments, especially in difficult situations (i.e., cesarean procedure and medical recovery) (Rafienia et al., 2008; Tamir, 2009). These automatic strategies could lead to discrepancies between what people feel and what they display, thus they are maladaptive and may determine psycho-pathological symptoms (i.e., anxiety, depression, psychotic symptoms; Mauss et al., 2007; Marszał-Wiśniewska and Nowicka, 2018). Future research should also assess whether the emotional regulation strategies would explain the relation between extraversion and postnatal psycho-pathological symptoms (i.e., anxiety, depression) on samples of new mothers who experienced the cesarean section.

Further, we measured postnatal depression 2 weeks after giving birth, and Peñacoba-Puente et al. (2016) assessed the postnatal depression 4 months after childbirth. The different questionnaires and scales used across studies to assess the personality traits or emotional disturbances could also explain these inconsistent findings. Finally, in their study Peñacoba-Puente et al. (2016) did not consider the type of birth as a potential moderator factor. The relations between personality traits and postnatal symptoms of anxiety and depression require further research.

Although this study enhances the literature, some limitations should be noted. Firstly, we used the self-reporting method to measure the main constructs in this study, meaning that the results could be distorted. A homecare visit to collect the women’s postnatal data, observations or more holistic assessments on various aspects of life (such as relation or routine) are necessary to enhance the current findings. Secondly, we relied on a short longitudinal design with a 2-week time interval between the assessments of postnatal anxiety and postnatal depression. Future studies should assess the interplay between postnatal anxiety and depression in order to provide a clearer image of the direction of the relations between these constructs, in longitudinal studies with multiple measurement points. Thirdly, we did not differentiate between clinical and non-clinical levels of postnatal anxiety or postnatal depression, although this would be necessary for better understanding the unique role of personality traits as risk factors in both mild and severe postnatal depression. Fourthly, we did not control the level of anxiety and depression before giving birth, and this limit should be considered in future longitudinal studies because previous literature showed that antenatal anxiety and depression are important risk factors for postnatal depression and anxiety. Finally, another limit of the study was that we did not account for the difference between emergency cesarean and planned cesarean section. Worries and anxiety in the postnatal period could be more accentuated after an emergency cesarean due to the fact that women do not know what to expect in the recovery period and for the future. Future studies are needed to investigate whether there are cesarean type procedure payments differences for postnatal anxiety and depression.

### Practical Implications

This study has theoretical and practical implications. Understanding how women’s personality traits relate to their emotional disturbance can help support (through psychological counseling) in specific areas to increase the likelihood of a positive postpartum period. Also, this work has helped to better understand psychological differences between groups of natural birth and cesarean birth. These results are especially important for those who play a role in supporting a mother’s decisions and experiences leading up to labor and delivery, such as doctors and nurses. The current findings add to the literature by providing better insight about how personality traits might foster a positive birth experience and a positive postpartum period. The results of this study can be used to develop psycho-educational and psychosocial interventions aimed at prevention, treatment and prevention of recurrence of postpartum affective disorder, considering the personality structure in order to achieve a more mature functionality and greater adaptability to the environment.

## Conclusion

Our study enhances the literature on the relation among personality traits, postnatal anxiety and postnatal depression during the postnatal period. We found that personality traits did not have a direct but a mediated impact on postnatal depression. Specifically, the relation between neuroticism and postnatal depression was explained by postnatal anxiety. Furthermore, we identified that the birth type may explain, as a moderator, the relations of personality traits and postnatal depression after 2 weeks from giving birth. This field may benefit from longitudinal studies to clarify potential bidirectional effects. In addition, future studies could evaluate other mechanisms that may explain the relations between personality traits and symptoms of anxiety and depression in the postpartum period.

## Data Availability

The datasets generated for this study are available on request to the corresponding author.

## Ethics Statement

The protocol of this study was approved by the Ethics Committee of Faculty of Psychology and Educational Sciences, Alexandru Ioan Cuza University and Medical Ethics Committee of the Clinical Hospital of Obstetrics and Gynecology of Cuza Voda, Iasi before beginning the study. All participants gave written informed consent in accordance with the Declaration of Helsinki.

## Author Contributions

MR and TC conceived and designed the study and collected the data. CB and LD-G analyzed the data. MR, CB, and LD-G wrote the manuscript.

## Conflict of Interest Statement

The authors declare that the research was conducted in the absence of any commercial or financial relationships that could be construed as a potential conflict of interest.
